# Responsible and adaptive robots in care home settings: an implementation framework analysis of a workshop with public and professionals

**DOI:** 10.3389/frobt.2025.1610329

**Published:** 2025-07-25

**Authors:** Andriana Boudouraki, Maria Waheed, Rafael Mestre, Aleksandra Landowska, Athina Georgara, Jayati Deshmukh, Lokesh Singh, Ayodeji O. Abioye, Nguyen Tan Viet Tuyen, Yi Dong, Shuang Ao, Dominic Price, Joel Fischer, Aislinn Gomez Bergin

**Affiliations:** ^1^ Mixed Reality Lab, School of Computer Science, University of Nottingham, Nottingham, United Kingdom; ^2^ Agents, Interaction and Complexity Group, School of Electronics and Computer Science, University of Southampton, Southampton, United Kingdom; ^3^ School of Computing and Communications, The Open University, Milton Keynes, United Kingdom; ^4^ Department of Computer Science, University of Liverpool, Liverpool, United Kingdom

**Keywords:** sensors, robotics, older adults, care work, healthcare, qualitative, implementation, social robots

## Abstract

As populations grow, research looks to emerging adaptive technologies for the urgent challenge in providing suitable care for older adults. Drawing on implementation science, we conducted a holistic examination looking at broader, contextual factors relating to the acceptability of robotics and sensor technologies in care homes. We held a workshop that brought together members of the public and researchers with experience in care home, to try such technologies and discuss their application in different care home scenarios. Using the NASSS framework, we examine acceptability through the angles of technology, condition, adopters, value proposition, organisation, wider context, and sustainability. While both groups of participants share concerns about the negative impacts of robotics on the quality of care, we also uncovered additional areas of further consideration relating to tensions between stakeholders and constraints around material resources, culture, processes and regulatory considerations.

## 1 Introduction

Robots are becoming increasingly common in our lives, offering physical and social assistance to create and enhance intelligent and adaptive living environments (Teng et al., 2022; Toh et al., 2016; Cone and Lambert, 2019). At the same time, many people now live longer, with the gap between the number of older adults needing care and the available care workforce widening (Sawik et al., 2023). Recent studies have explored how robots can assist in care settings (e.g., Papadopoulos et al., 2020; Koh et al., 2021) but these initiatives have so far failed to create meaningful impact, failing to capture the broader, complex and contextual realities of care work. Many such studies have narrowly focused on specific technologies or stakeholder groups (Trainum et al., 2024). In contrast, our workshop brought together both care professionals and members of the public to reflect on the combined use of emerging robotic and sensor technologies, exploring not just acceptability but also how such systems might realistically integrate into care home environments.

Developments in assistive robotics and sensor technologies offer increasingly interactive and customizable solutions, with face and voice recognition, speech interaction, and context-aware responses (Guemghar et al., 2022; Koh et al., 2021). We might imagine robots, using sensors and Artificial Intelligence (AI) to deduce information about the users and adapt their ways of acting and speaking accordingly (Cheresharov et al., 2024; Irfan et al., 2024).

However, we first need to critically examine what is truly needed, and understand the context in which solutions would be implemented. Initiatives promoting ethical and responsible innovation (e.g., Portillo et al., 2023; Stilgoe and Guston, 2016) highlight the importance of anticipating societal impact, engaging with stakeholders from the early stages of research and understanding the broader context. Involving stakeholders in the design process and addressing privacy and user autonomy concerns are also essential for fostering trust and acceptance (Sharkey and Sharkey, 2012; Van Wynsberghe, 2020). Within care homes, this involves understanding the needs of several types of stakeholders, like residents, residents’ families, carers, broader staff and management, including volunteers (Servaty et al., 2020; Koh et al., 2021; Guemghar et al., 2022).

In addition, to ensure that technology brings benefits rather than barriers, we must take into account multiple factors, including practical matters such as space and material resources, familiarity with technology, organisational structures and legal concerns (Guemghar et al., 2022; Koh et al., 2021). For example, using robots in care settings results in some additional risks and challenges (Hung et al., 2022) like cross-contamination due to using robots (Bradwell et al., 2020) and overtrust in robots (Borenstein et al., 2018). In order to capture these contextual factors, our analysis is guided by the NASSS (Non-adoption, Abandonment, Scale-up, Spread, Sustainability) framework, which draws on implementation science and has been specifically designed to examine concrete and multifaceted considerations relating to the adoption of technology in healthcare organisations (Greenhalgh et al., 2017).

Our work explores the challenges and opportunities that exist in this domain, with particular focus on stakeholders’ concerns and contextual factors. We reported on a workshop we conducted with members of the public and professionals with work experience within care homes. Our aim was to collect insights on the acceptability and suitability of robotic and sensor technologies, through understanding the stakeholders’ concerns and the broader practical, organisational and regulatory factors. We analysed and presented our findings, using the NASSS framework (Greenhalgh et al., 2017), which allows us to examine acceptability by looking at seven domains: technology, condition, adopters, value proposition, organisation, wider context, and sustainability. Beyond insights into the factors affecting the succesfull adoption of sensors and robots in care homes, we contribute by demonstrating how the NASSS framework can be used to examine qualitative data from stakeholder engagement research and discuss the limitations of this approach.

## 2 Methods

Ethical approval for this study was granted by the University of Nottingham’s, Computer Science Research Ethics Committee (Reference: CS 2024R11).

### 2.1 Participants

The workshop was attended by older adults from the general public (n = 4, 3 men and 1 woman, average age = 70) and professionals with experience conducting research in care homes (n = 4, 2 men, 2 women).

### 2.2 Workshop

The two groups (public and professionals) were divided into separate tables, in order to gain distinct insights on the perspectives of each group and prevent one perspective from dominating the discussion.

First, we demonstrated a variety of scientific-grade and consumer sensors, inviting participants to try them out. We showed EEG sensors (Muse 2 and Emotiv Epoch), funcational near-infrared spectroscopy (fNIRS) devices (Artinis Brite, OctaMon, and Mendi) and the Tobii Pro eye tracker, the Shimmer GSR sensor, and a Fitbit smartwatch. We selected these as they capture physiological data that might be relevant in a care setting, and in order to show participants the range of sizes and forms that sensors can take.

Second, we demonstrated various robots, including Pepper, MiRo, Temi and Boston Dynamics’ Spot and explained their capabilities and potential applications. The robots were selected to give participants an understanding of the variety of sizes, appearance (e.g., anthropomorphic, animal-like, computer-like) and capabilities (e.g., mobile, stationary, with screen or voice interface, etc).

The final activity focused on brainstorming scenarios in which these technologies might be used in a care home. To start the conversation, participants were shown a scenario of a dining room in a care home—a use case already implemented in restaurants, and therefore potentially viable (Jang and Lee, 2020). We showed them a diagram and described how robots might assist a human server in taking orders and carrying food to and from the kitchen (see Supplementary Material). They were then asked to discuss how realistic and desirable they found it and suggest alternative use cases where this technology might be more useful and acceptable. We used prompts to guide this discussion, covering topics such as the physical environment, everyday activities, the people involved, clinical and regulatory issues, safety and research directions (see Supplementary Material). These were designed to provide us with a concrete and holistic understanding of the practical realities of care homes and care work.

### 2.3 Analysis

Each table was facilitated by one researcher, with 3 observers taking notes. Audio recordings of the discussions were collected to complement any gaps or unclear notes. We digitised our notes and analysed this data through framework analysis; a procedure for analysing qualitative data, using pre-defined frameworks to categorise data into meaningful groups (Goldsmith, 2021).

We based our analysis on the NASSS framework (Greenhalgh et al., 2017); which specifically targets the long-term suitability of technologies in healthcare organisations. The framework categorizes factors into the domains of: technology, condition, adopters, value proposition, organisation, wider context, and sustainability).

Following the framework analysis protocol, we coded the data using labels that characterized the issues described. Then, the data items, together with their codes, were categorised within the aforementioned NASSS domains. Up to this point, each of the facilitators coded and categorised the data corresponding to notes taken from the table they were facilitating, as their role in facilitation allowed them to better contextualise the notes. Finally, the two facilitators and one more researcher, in a collaborative process, re-examined the codes together and identified overarching themes in each domain. We also specifically looked for similarities and differences in the issues raised by each group.

## 3 Results

The first four domains of the NASSS framework (condition, technology, value proposition and adopters) were represented in discussions with both groups, while the organisation domain was only discussed by the professionals. Our data did not fit the domains of wider context and sustainability, but implications are discussed in Section 4.

### 3.1 Condition

Condition in the NASSS framework refers to the illness or condition for which a technology is designed. Regarding care homes, we look at the physical and social care needs of older adults that robotics and sensors might address.

When presented with the technology and potential scenarios, both groups opposed the idea of robots replacing any of the social aspects of care.

The professionals were highly critical of the idea of robots performing any of the social aspects of care work. They deemed this an essential and highly skilled aspect of their work; automating it would reduce the quality of care and devalue their skills. When asked whether sensors might be useful for deducing the needs of non-verbal residents, they reported that skilled carers are able to understand how a resident is feeling and how to best approach them. When asked whether robotics might be helpful in serving food, they noted that feeding is an intricate and social activity; an opportunity for the carer to build rapport and check in with the resident. In addition, carers are trained in making decisions about whether to prioritise the resident’s health or personal freedom with regards to their eating options on a case-by-case basis. These moments, when the carer is physically and socially interacting with the residents, are at the core of what care work entails, and professionals felt that they should be left to humans.

Instead, the professionals suggested that robotics and sensors would be valuable in automating tasks that do not involve the residents directly, such as housekeeping. They brought up the idea of robots working ‘backstage’, carrying items (e.g., bedsheets) or helping avoid cross-contamination by carrying equipment without coming into contact with people or areas that need to remain clean. They also proposed that robotics and sensors might be valuable in helping identify whether a resident has fallen or needs assistance, to reduce false alarms.

Members of the public were less critical of robots in social roles but still expressed concerns about the care being of equal quality. For example, whilst they could imagine that a robot, rather than a person, might pick up their plates after eating, they noted that a human would ask them if they want more food. Through this directed and personalised attention, human carers provide meaningful care even in seemingly mundane and practical tasks.

Some members of the public were open to the idea of robots taking the role of pets, to provide a sense of companionship. Others, whilst not wanting a ‘pet robot’ themselves, understood why other people might be open to this, expressing that it is a matter of personal preference.

Overall, both sides expressed the sentiment that robotics and sensors should support human care work, rather than replace it.

### 3.2 Technology

The domain of technology looks at aspects of the technology itself (how it functions, what it does, what is required to make it work).

Both the public and professionals expressed concerns about the reliability of their robots (their capacity to perform as expected), and remarked on their potential intrusiveness. They were curious about whether robots could discern between different users and be customisable to different users’ preferences and needs. The professionals were more focused on practical aspects of what the robots could and could not do (such as maintaining hygiene, or avoiding cross-contamination) and whether maintenance of the robot itself would increase their workload. The public, instead, focused on aspects relating to the user experience, appearance and effect of having robots in their day-to-day lives.

Discussions during the sensors demonstration focused on both how it felt to wear them and the data that was being collected. The public group focused more on comfort, design, wearability, and who the data was shared with. The professionals focused on the reliability and accuracy of the data. Both groups felt that wearable sensors are more suitable, as they are potentially less intrusive, smaller, and more comfortable to use; however, they also raised concerns about their reliability and emphasised the importance of validating wearable devices against scientific-grade sensors.

### 3.3 Value proposition

Value proposition refers to the added benefit that this technology brings to the organisation and its stakeholders.

Both groups felt that robotics and sensors might alleviate some of the workload of the care home staff. As explained above, robotics could be employed to assist with housekeeping and sensors might allow caregivers to monitor residents more efficiently.

However, the professionals noted that potential costs are also involved. Namely, they highlighted additional resources and training required to support the use of robots. Care homes tend to have a frequent turnover of staff, so the cost of training someone might not be worthwhile if they leave soon. They also wondered whether operating the robots might burden staff with additional work, such as overseeing the robots and cleaning them. Although they saw potential in robots in helping to avoid cross-contamination by carrying certain items, they were also concerned that the robots themselves might become a hygiene hazard if not cleaned properly. In addition, when it comes to using sensors as a form of deducing residents’ conditions, professionals showed reluctance to trust such data; if it requires staff to perform additional evaluations themselves to validate it, the technology might be redundant and therefore less valuable.

### 3.4 Adopters

The adopters domain includes the thoughts and feelings of adopters and members who are going to be affected by the technology.

Both groups shared concerns about the use of sensor data. Both worried that the data collected by the sensors might be misused or misinterpreted. They questioned whether physiological data can really reveal how people are feeling, and whether the staff or robots can interpret the data correctly to make decisions about how to treat the residents. Participants representing members of the public were particularly reluctant to accept the validity of such data and claimed that they would need to see a proof-of-concept before conceding to use it.

Both groups also expressed concerns about surveillance. Professionals discussed that some might not want their physiological data to be transparent or available to others. Discussion the use of data relating to stress levels, they noted that people have different thresholds of manageable stress and feared that sharing such data could put their jobs at risk or result in more unpleasant working conditions. Although we probed them to discuss useful applications of their physiological data, such as helping them allocate work tasks more efficiently, they were adamantly against this due to distrust of care home management. They claimed that management have little understanding of care work, and explained that any data on the workers would be used to reduce costs rather than improve the quality of care.

The members of the public noted that the collection of sensor data should be done with consent and questioned whether it would be ethical to use on residents who are unable to give informed consent. They also reported that they would only be willing to wear sensors in very specific situations (such as for serious mental illness) rather than for regular use.

Both groups expressed similar concerns around the capability of this technology to allow for individual differences. Professionals noted that care home residents might differ in how familiar and comfortable they are with technology—their concern mainly focusing on the impact this will have on the residents’ experience. They noted that some care home residents are nervous around technology, or suffer from anxiety which robotic technology might exacerbate. Members of the public, confirming this belief, showed variability in how open they were to the idea of interacting with robots or whether they were open to the idea of keeping a robot pet as a companion.

Beyond this, professionals discussed whether the use of robots in a care home might result in residents developing emotional relationships with those robots and the impact this might have on them (especially if the robots eventually change or malfunction).

In addition, reflecting on the ideas mentioned in Section 3.1, professionals expressed worry around potential de-skilling effects. These participants were not simply worried about the loss of jobs, but most importantly about the devaluation and undermining of care work. They viewed the work of human carers as highly skilled and intricate, and felt that the technological alternative would provide a worse, less meticulous, form of care (lowering the standards for this work overall).

Echoing these feelings, participants from the public also reported that they preferred to be cared for by people rather than by technology. Whilst some of them were open to the idea of robots offering them companionship and an opportunity for ‘social’ interaction, they generally did not want to be dealing with technology all the time.

### 3.5 Organisation

This domain describes the structure and management of the organisation, including the processes, regulations and people involved with deploying the new technology.

We only obtained data on this from the professionals group. Their discussions highlighted that there are vast organizational differences across care homes in the United Kingdom. The participants described care homes of different sizes (both in terms of the physical space as well as the structure). Some care homes are part of a wider organisation, managed centrally, while others are independent. There is variety in how staff’s responsibilities are arranged, with some homes having highly specialised staff (e.g., a person in charge of leisure activities) and others where staff cover many different roles (e.g., activities, healthcare, cleaning). There is no standardisation in what care entails and how it is operationalised. Some care homes involve residents in day-to-day housekeeping and cooking activities, as a way of keeping them active and engaged, while in others the residents are not expected to do any work.

In terms of dealing with the technology, the participants again noted that care homes have different set-ups (e.g., different software for data management) and limited IT resources. Introducing a new technology would involve training members of staff on how to operate it, as there is generally no dedicated IT specialist.

Discussions around the organisational reality of care homes also led participants to question who would bear the responsibility of managing the robots. As mentioned above, they noted that care home owners and management often have little or no background in care. At the same time, it is those stakeholders who bear the legal responsibility if something goes wrong in the care home. This might result in a mismatch between the technology that gets deployed (and how it is managed) and the reality of what staff and residents actually need.

## 4 Discussion

Here we discuss each of the NASSS framework domains in relation to previous literature and highlight direction where more research would be beneficial.

### 4.1 Condition

The caring role was viewed with significant respect by both professionals and the public. The public appreciated the additional gestures and care provided by humans over what they would anticipate from a robot, while professionals valued the time spent with those being cared for as an opportunity to establish relationships, thus also providing benefits to the carers. These are not surprising results considering the preference for more conventional care where needs are complex (Warmbein et al., 2023). This underscores the limitations of technologies in achieving this level of intricate sophistication (Sawik et al., 2023). Increased familiarity and tailored approaches to individual needs are needed for the delivery of care that more accurately simulates the personalisation offered by human carers but also contribute to building trust in these systems (Koh et al., 2021; Guemghar et al., 2022).

### 4.2 Technology

Concerns raised in our study align with the literature; the professionals’ primary focus is on whether the technology can be relied upon to perform as expected and continue functioning (Koh et al., 2021). Conversely, the public is concerned with how these technologies might impact their daily lives (Papadopoulos et al., 2020). While familiarity can assist in mitigating these concerns (Papadopoulos et al., 2020), many individuals’ exposure to robots and AI is primarily through media representations, evoking fear and uncertainty (Liang and Lee, 2017). Additionally, there are concerns regarding accuracy and appropriate use of data collected through sensors. These concerns need to be addressed both technically and socially to ensure success. The potential of adaptive and responsive systems lies in their ability to demonstrate successful applications that can account for the substantial diversity of experiences within the care home environment.

### 4.3 Value proposition

Both groups felt that the robots would be well-suited to more menial tasks. This may reflect a desire to keep robots out of the way, manifesting the distrust people feel regarding their reliability. The literature highlights that carers are often concerned about being replaced and adoption is more successful where systems are seen as complementary (Sawik et al., 2023; Servaty et al., 2020). Whilst direct costs are often discussed, our workshop highlighted often-hidden or unexpected costs such as training, hygiene, and ongoing monitoring, which the design of future technologies needs to take into account. Additionally, there may be unforeseen or extra costs associated with the benefits, such as additional work.

### 4.4 Adopters

Implementation may be more successful where more stakeholders are involved, such as staff, residents, family members, carers, volunteers, along with care home managers and owners. These different perspectives and their impact on decisions about the design and implementation of systems, are important to consider. For example, if more sophisticated data is being collected there may be concerns about how this is being used, e.g., for surveillance, thus reducing older adults’ autonomy (Sawik et al., 2023). Recognising the different perspectives and ensuring these are accounted for can provide opportunities for these concerns to be addressed and mitigated either technically or in more social ways such as through reassurance.

### 4.5 Organisations

Discussions with professionals also suggested the disconnect between organisational business-oriented priorities and the realities of frontline care which could lead to technologies being acquired that are not implementable in practice. There was also significant variability across care environments in several aspects, including space, staff responsibilities, daily activities and approaches to care. This indicates that responsive and adaptive systems are potentially a way to overcome some of these barriers by offering systems that can allow for more diversity in not just interactions with humans but also in infrastructure and deployment.

### 4.6 Limitations: wider context and embedding and adaptation over time

Our participants did not explicitly discuss issues pertaining to the wider context or long-term adoption. Discussions with the professional group pointed towards wider contextual factors, including legislations and regulatory bodies but did not provide concrete insights. Indeed literature mentions the need for greater clarity in these areas (Sawik et al., 2023; Koh et al., 2021; Papadopoulos et al., 2020). This points to a limitation in our approach—of applying the NASSS framework on workshop data—and highlights the importance of holistic and multidisciplinary work. Addressing these domains might require discussions with other types of stakeholders, such as managers, who may have more insight on these areas, but also more in-depth scoping of the care home sector, collaboration with organisation science or policy practitioners, and longitudinal studies of technology adoption in that setting. While familiarity could mitigate many concerns, this workshop and much of the literature are restricted by being of very short duration (Koh et al., 2021; Guemghar et al., 2022; Papadopoulos et al., 2020; Servaty et al., 2020). What is clear, from our research and the literature, is that involvement and co-production can facilitate more sustainable development across not only the design of the technologies but also the social infrastructures and human interoperability that are required.

### 4.7 Recommendations and future work

The NASSS framework, as demonstrated above, allows us to identify context-specific and tangible dimensions of technology acceptability. Through this lens, the (non-) adoption of technology is not a matter of (non-) users’ personal ethics but about multifaceted and practical considerations. Participants were open to robotic and sensor technology that could solve their problems (e.g., detect falls), but resistant due to specific negative impacts (additional work, misuse, worsening of care standards). As such, a responsible approach to the implementation of adaptive robots in case settings involves designing with a clear understanding of the needs and capacities of those settings.

Our present work highlighted several areas where the implementation of sensors and robotics in care homes needs to be considered more carefully. From practical (storage), organisational (maintenance) and safety (cross-contamination) concerns to issues around data privacy, future work should address each area in more depth with sensitivity to how each issue fits within the broader context and intersects with the needs and preferences of other stakeholders. As discussions around the appropriate use of sensor data were predominant in our participants’ discussions, the ongoing work of the authors is concerned with understanding what levels of robot adaptability—through sensor data—are acceptable to the stakeholders of care homes, and what forms of sensor data may be used to achieve it. Our work aims to explores this through Wizard of Oz experimental trials, as well as qualitative engagement with interviews and workshops.

Beyond this, it is imperative that we explore the role that responsible and adaptive robots might have within care homes with consideration of different adopters, organisational factors, and legislation. Whilst acceptance of technology is a widely acknowledged area of research, it only goes so far in addressing the complexities of settings such as care homes, where the tensions and constraints can move beyond what can be seen within the environment and encompass culture, processes, and contexts that require in-depth research to uncover. As we have shown, there are shared but also varied opinions between the stakeholders. Researchers should consider how to address these groups in a multidisciplinary capacity, equipping the project team with the tools to understand multiple domains upon which responsible and adaptive robot implementation is dependent. Moreover, we advocate for broader engagement with less obvious stakeholder—care home managers, volunteers, cleaning staff, IT technicians, patients’ relatives, and others. This is particularly important given that tensions may arise between groups such as managers and care workers, due to differing understandings of care needs and the potential impact of robotics in this context. Other care home staff, like cleaning personnel or IT technicians, can offer distinct yet essential perspectives, as their workloads and responsibilities may be significantly affected by the introduction of robots—potentially revealing important prerequisites or opportunities.

## 5 Conclusion

We explored the potential introduction of responsive and adaptive robotics into care homes by engaging with both members of the public and professionals with experience in care. Building on prior work on implementation science, we employed the NASSS framework to structure our analysis and extended this by introducing the concept of adaptive systems, where sensor technologies are integrated into robotic platforms, through a series of interactive demonstrations. The NASSS framework enabled us to systematically examine insights across its seven domains, revealing both shared and divergent perspectives among stakeholders. Our findings suggest that while some domains such as technology, condition, and value proposition tend to generate more explicit reflections and easily shared experiences, others, particularly the wider context and the processes of embedding and adaptation over time, remain more implicit, although not less complex. These less articulated domains are also important and may require more deliberate, participatory, and iterative methods to fully explore and address in future research and implementation efforts.

## Data Availability

The raw data supporting the conclusions of this article will be made available by the authors, without undue reservation.
