# Supplementary material for: Responsible and adaptive robots in care home settings: an implementation framework analysis of a workshop with public and professionals
Source: Front Robot AI. 2025 Jul 25;12:1610329. doi: 10.3389/frobt.2025.1610329 (PMC12331501; doi:10.3389/frobt.2025.1610329)
Supplement: Supplementary file 1 [file DataSheet1.pdf]

Supplementary Material

1 WORKSHOP SCENARIO AND DISCUSSION PROMPTS

The diagram shown in Figure S1 was printed on A4 paper and shown to the participants as a visual aid to guide our presentation of a potential scenario and initiate the discussion. In addition to showing this image, we described how human and robotic staff might work together in a dining room to serve and collect food from the tables.

1.1 Dining Room Scenario

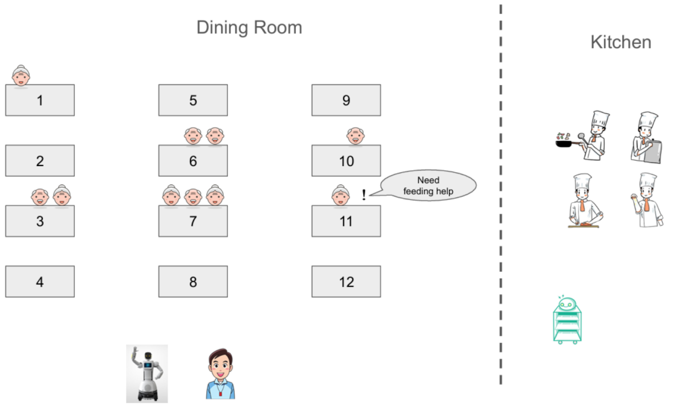

Figure S1. The figure was shown to participants during the discussion part of the workshop. It shows an abstract representation of a dining room and kitchen areas, with human and robot staff.

1.2 Discussion Prompts

The following questions were used by the facilitators to guide the workshop discussions.

- Environment – how would robots and sensors fit, not fit.
- Daily life – what is the impact on routines and interactions?
- People – what is the impact on staff, volunteers, residents, visitors?
- Clinical, regulatory, legal – what considerations are there? e.g., data
- Safety and risk – what considerations are there? E.g., falls
- Functionality – evaluate needs, challenges, opportunities. What would you like them to do or not do?
- Research – what should we consider when doing research in these spaces?
